# Risk factors of conversion to hand-assisted laparoscopic surgery or open surgery in laparoscopic liver resection: a multicenter prospective study (HiSCO-08)

**DOI:** 10.1038/s41598-025-34013-3

**Published:** 2025-12-26

**Authors:** Ko Oshita, Michinori Hamaoka, Tsuyoshi Kobayashi, Takashi Onoe, Tomoyuki Abe, Toshihiko Kohashi, Koichi Oishi, Daisuke Takei, Tomoyuki Akita, Hideki Ohdan

**Affiliations:** 1 Department of Gastroenterological and Transplant Surgery, Graduate School of Biomedical and Health Sciences, Hiroshima University, Hiroshima, Japan; 2 Department of Gastroenterological, Breast and Transplant Surgery, Hiroshima Prefectural Hospital, Hiroshima, Japan; 3 Department of Gastroenterological Surgery, Chugoku Cancer Center, Kure Medical Center, National Hospital Organization, Hiroshima, Japan; 4 Departmet of Gastroenterological Surgery, Higashihiroshima Medical Center, National Hospital Organization, Hiroshima, Japan; 5 Department of Hepato-Biliary-Pancreatic Surgery, Hiroshima City North Medical Center Asa Citizens Hospital, Hiroshima, Japan; 6 Department of Surgery, Chugoku Rosai Hospital, Hiroshima, Japan; 7 Department of Surgery, Onomichi General Hospital, Hiroshima, Japan; 8 Department of Epidemiology, Graduate School of Biomedical and Health Sciences, Infectious Disease Control and Prevention, Hiroshima University, Hiroshima, Japan; 9 Department of Gastroenterological Surgery, Hiroshima Prefectural Hospital, 1-5-54 Ujina-kanda, Minami-ku, Hiroshima, 734-8530 Japan

**Keywords:** Conversion, Hand-assisted laparoscopic surgery, Laparoscopic liver resection, Open liver resection, Repeat liver resection, Cancer, Diseases, Gastroenterology, Medical research, Oncology, Risk factors

## Abstract

Laparoscopic liver resection (LLR) is widely accepted; however, no prospective study has identified risk factors for conversion to open surgery. This multicenter prospective study aimed to identify risk factors for conversion to hand-assisted laparoscopic surgery or open liver resection and evaluate the impact of conversion on perioperative outcomes in LLR. From June 2020 to May 2024, patients scheduled for wedge resection or left lateral segmentectomy for solitary liver tumor < 5 cm were enrolled across seven institutions. A laparoscopic approach was employed for all patients. The primary endpoint was the identification of risk factors for conversion. As secondary endpoints, we compared perioperative outcomes between LLR and conversion. Among 199 patients, 172 (86.4%) completed LLR, while 27 (13.6%) required conversion. Multivariate analysis identified third or subsequent liver resection (*P* < 0.001) and male sex (*P* = 0.029) as significant risk factors for conversion. Conversion was significantly associated with longer operative time (*P* = 0.001), greater blood loss (*P* < 0.001), decreased implementation of the Pringle maneuver (*P* = 0.013), and prolonged hospital stay (*P* = 0.001). These findings highlight that conversion negatively impacts short-term outcomes. Awareness of these risk factors may support better surgical planning and risk stratification for patients undergoing LLR.

**Supplementary Information:**

The online version contains supplementary material available at 10.1038/s41598-025-34013-3.

## Introduction

Since the feasibility and favorable indications for laparoscopic liver resection (LLR) were stated at the first international consensus conference in Louisville in 2008^1^, LLR has become increasingly accepted and performed worldwide^2,3^. Numerous studies have demonstrated the effectiveness of LLR compared with conventional open liver resection (OLR) in terms of perioperative outcomes, including reduced blood loss, fewer postoperative complications, and shorter hospital stays^4–6^.

However, in some cases, conversion to hand-assisted laparoscopic surgery (HALS) or OLR is necessary for oncological and safety reasons^7–9^. Common reasons for conversion include bleeding, adhesions, and oncological concerns, and it is well established that the perioperative outcomes are inferior to those of successfully completed LLR^9,10^. Previous studies have identified several patient and tumor-related risk factors for conversion^11–14^. However, these studies were retrospective analyses of selected patients, often excluding cases with factors considered unsuitable for LLR such a history of open superior abdominal surgery or liver resections preoperatively, and the results may have been significantly affected by selection bias. As LLR continues to become more widely adopted, practical identification of risk factors for conversion is increasingly important to ensure more patients can benefit from its advantages.

Wedge resection and left lateral sectionectomy (LLS) are commonly recommended minor liver resections for benign tumors and small malignant tumors. Therefore, a laparoscopic approach was employed for all patients scheduled for wedge resection or LLS for liver tumor regardless of the expected difficulty of the laparoscopic surgery in this prospective study. In this study, we aimed to investigate the risk factors for conversion and evaluate their impact on perioperative surgical outcomes in LLR.

## Methods

### Study design and participants

This multicenter prospective study was conducted at seven institutions affiliated with the Hiroshima Surgical Study Group of Clinical Oncology (HiSCO). The study protocol was approved by the institutional review board of Hiroshima University, Japan, and each participating hospital (Approval No. C2020-0294). This study was registered with the University Hospital Medical Information Network (Registered No. UMIN000039957) and conducted in accordance with the Declaration of Helsinki. Written informed consent was obtained from all patients.

### Data collection

Clinical data collected included patient characteristics, comorbidities, preoperative blood examination results, tumor characteristics, history of abdominal disease and surgery, details of previous liver resection, surgeon’s experience with LLR, operative findings, and postoperative outcomes. Primary liver malignancies included hepatocellular carcinoma, intrahepatic cholangiocarcinoma, combined hepatocellular and cholangiocellular carcinoma, and malignant lymphoma. Liver resections were performed according to the 2000 Brisbane terminology^15^, and the difficulty of liver resections was graded using the Iwate criteria^2^. Posterosuperior segments included segments 1, 4a, 7, and 8. Postoperative complications were categorized according to the Clavien–Dindo (CD) classification, and complications graded as CD ≥ 3 were considered major. Post-hepatectomy liver failure (PHLF) and bile leakage were assessed according to the International Study Group of Liver Surgery definitions.

### Endpoints

The primary endpoint was risk factors for conversion. Conversion is a procedure requiring a laparotomy, excluding laparotomy for specimen extraction or trocar placement. In this study, it was defined as a switch to HALS or OLR. Secondary endpoints were surgical outcomes, including operative time, blood loss, intraoperative transfusion, and implementation rate of the Pringle maneuver, postoperative complications, mortality, and postoperative hospital stay.

### Inclusion and exclusion criteria

The indications for LLR in this study were based on the Louisville consensus, with the addition of segments 1, 7, and 8^1^. The eligibility and exclusion criteria were as follows.

Eligibility criteria:

(1) Scheduled for wedge resection or LLS for a solitary liver tumor. (2) A tumor < 5 cm in size located in the periphery of the liver. (3) Eastern Cooperative Oncology Group performance status of 0–1. (4) Age ≥ 20 years at the time of informed consent. (5) No severe hematological, liver, renal, or cardiopulmonary dysfunction.

Exclusion criteria:

(1) Required biliary duct reconstruction or revascularization. (2) Required simultaneous resection of organs other than the gallbladder.

### Surgical procedure

Indications for liver resection were determined by a multidisciplinary team at each institution, including surgeons, hepatologists, radiologists, and pathologists, depending on tumor status, liver function, and patient status. The surgical team included surgeons experienced in more than 50 cases of liver resection and laparoscopic procedures, respectively.

A laparoscopic approach was employed for all enrolled patients in this study. The basic policies and procedures for LLR have been previously detailed^16^. Briefly, a 12 mm port was placed using the open method, and four ports were placed depending on the tumor’s location. Intraoperative ultrasonography was used to confirm tumor extent and define the transection plane. Parenchymal transection was performed using a Cavitron ultrasonic surgical aspirator and bipolar forceps. The Pringle maneuver was applied in principle, except in cases of severe adhesions around the hepatoduodenal ligament.

To ensure safety and curability, the following criteria for conversion were determined by consensus among surgeons from the participating institutions:

(1) Adhesion separation deemed impossible or expected to exceed 120 min. (2) Expected blood loss exceeding 1000 mL. (3) Pringle maneuver duration expected to exceed 120 min. (4) Difficulty in achieving complete tumor resection. (5) Surgeon’s determination that continuing laparoscopic surgery was unsafe for any reason.

If continuation of LLR was considered difficult, conversion to HALS was considered to restore the surgical procedure. Conversion to OLR was performed if laparoscopic surgery was unsafe or compromised oncological curability. In conversion to HALS, an 8–10 cm incision was added, and a hand port was introduced to assist in subsequent procedures. For OLR conversion, a midline or L-shaped incision was made, extending from the umbilical port.

Patients were followed up for at least 90 days after surgery, and then follow-up and treatment continued depending on the disease.

### Statistical analysis

Continuous variables were expressed as median (interquartile range [IQR]) and categorical variables as numbers (percentages). Continuous variables were tested using Student’s t-test for comparisons between two groups and the Tukey–Kramer test for three groups. Categorical variables were tested using the chi-squared test or Fisher’s exact test, with Bonferroni correction performed for comparison between three groups. The cut-off value of the number of liver resections for the risk of conversion was determined using receiver operating characteristic (ROC) curve analysis. Univariate and multivariate analyses were performed using a logistic regression model, and the relevant univariate variables (*P* < 0.05) were included in the multivariate analysis. A P-value < 0.05 and a Bonferroni correction P-value < 0.017 were considered statistically significant. All statistical analyses were performed using JMP version 18.0 (SAS Institute Inc., Cary, NC, USA).

### Sample size

The required sample size for univariate analysis was first calculated^17^ and then converted to the sample size required for multivariate analysis based on the multiple correlation coefficient^18^. Assuming a conversion rate of 15%, an unadjusted odds ratio of 4.0, and a factor prevalence of 0.2–0.45 in both the conversion and pure LLR groups, the required sample size for univariate analysis was estimated to be 20–23 cases in the conversion group and 114–131 cases in the pure LLR group. Next, assuming a moderate correlation among factors and setting the multiple correlation coefficient to ρ = 0.4, the required sample size for multivariate analysis was calculated as 24–28 cases in the conversion group, 137–158 cases in the pure LLR group, and 161–186 cases in total. Considering an expected dropout rate of 7–10%, the target sample size was set at 200 cases.

## Results

### Patient characteristics

**Fig. 1:**
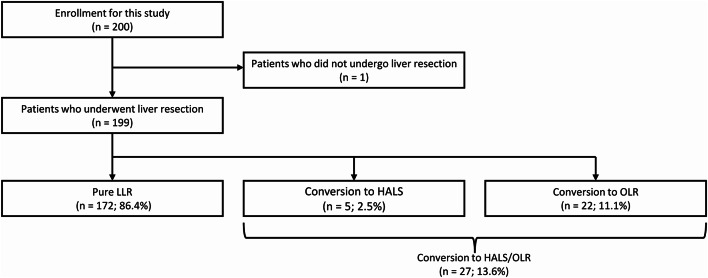
Study flowchart of this prospective study. HALS, hand-assisted laparoscopic surgery; LLR, laparoscopic liver resection; OLR, open liver resection.

Between June 2020 and May 2024, 200 patients were registered at seven facilities after eligibility assessment; one patient who did not undergo liver resection was excluded. Consequently, 199 patients were included in the analysis. Among the patients, 172 (86.4%) underwent pure LLR, 5 (2.5%) converted to HALS, and 22 (11.1%) converted to OLR, with a conversion rate of 13.6% (Fig. 1).

The patient characteristics are shown in Table 1. The median age was 75 years (IQR, 70–80 years); 144 patients were male (72.4%), and 55 were female (27.6%). Among the patients, 114 (66.8%) underwent surgery for primary liver malignancy; 56 (28.1%) had liver metastasis, and 8 (4.0%) had benign tumors.

**Table 1:** Patient characteristics.

Variables	Whole cohort (*n* = 199)	Pure LLR (*n* = 172)	Conversion to HALS/OLR (*n* = 27)		*P*-value
Age (years)	75 (70–80)	75 (69–80)	75 (71–81)		0.935
Sex (Male/Female)	144 (72.4%):55 (27.6%)	119 (69.1%):53 (30.8%)	25 (92.6%):2 (7.4%)		0.012
Body mass index (kg/m^2^)	23.8 (21.5–26.4)	23.7 (21.4–26.3)	24.6 (21.7–27.0)		0.222
HBV positive	24 (12.1%)	19 (11.0%)	5 (18.5%)		0.268
HCV positive	56 (28.1%)	50 (29.1%)	6 (22.2%)		0.462
Diabetes	69 (34.7%)	58 (33.7%)	11 (40.7%)		0.476
Hypertension	119 (59.8%)	100 (58.1%)	19 (70.3%)		0.228
Cirrhosis	55 (27.6%)	48 (27.9%)	7 (25.9%)		0.831
Diagnosis					0.316
Primary liver malignancy	134 (67.3%)	113 (65.7%)	21 (77.8%)		
Liver metastasis	56 (28.1%)	50 (29.1%)	6 (22.2%)		
Benign tumor	9 (4.5%)	9 (5.2%)	0 (0.0%)		
Total bilirubin (mg/dL)	0.8 (0.5–1.1)	0.8 (0.5–1.1)	0.9 (0.7–1.1)		0.298
Albumin (g/dL)	4.0 (3.8–4.3)	4.1 (3.8–4.4)	3.9 (3.7–4.0)		0.007
Prothrombin time activity (%)	97 (88–104)	98 (91–105)	90 (82–98)		0.597
Child-Pugh classification A/B	192 (96.5%)/7 (3.5%)	165 (95.9%)/7 (4.1%)	27 (100%)/0 (0.0%)		0.103
Platelet count (10^9^/L)	174 (132–212)	175 (138–211)	146 (109–224)		0.618
Aspartate aminotransferase (IU/L)	24 (20–34)	24 (20–32)	33 (21–35)		0.779
Alanine aminotransferase (IU/L)	19 (14–28)	19 (14–28)	22 (16–29)		0.654
ICG R15 (%)	12.6 (7.9–18.3)	11.5 (7.7–17.6)	16.4 (9.5–21.2)		0.209
Creatinine (mg/dL)	0.84 (0.69–1.00.69.00)	0.84 (0.70–0.98)	0.84 (0.69–1.08)		0.917
Tumor size (mm)	15 (10–21)	15 (10–21)	15 (10–22)		0.679
Tumor location Posterosuperior segments	52 (26.1%)	44 (25.6%)	8 (29.6%)		0.656
Difficulty score Low/Intermediate	126 (63.3%)/73 (36.7%)	108 (62.8%)/64 (37.2%)	18 (66.7%)/9 (33.3%)		0.698
Previous abdominal surgery	146 (73.4%)	120 (69.8%)	26 (96.3%)		0.004
Previous upper abdominal surgery	95 (47.7%)	71 (41.2%)	24 (88.9%)		< 0.001
Initial/Repeat liver resection	119 (59.8%)/80 (40.2%)	116 (67.4%)/56 (32.6%)	3 (11.1%)/24 (88.9%)		< 0.001
Third or subsequent liver resection	32 (16.1%)	16 (9.3%)	16 (59.3%)		< 0.001
Surgeon’s experience with LLR < 50 cases	133 (66.8%)	112 (65.1%)	21 (77.8%)		0.194

**Fig. 2:**
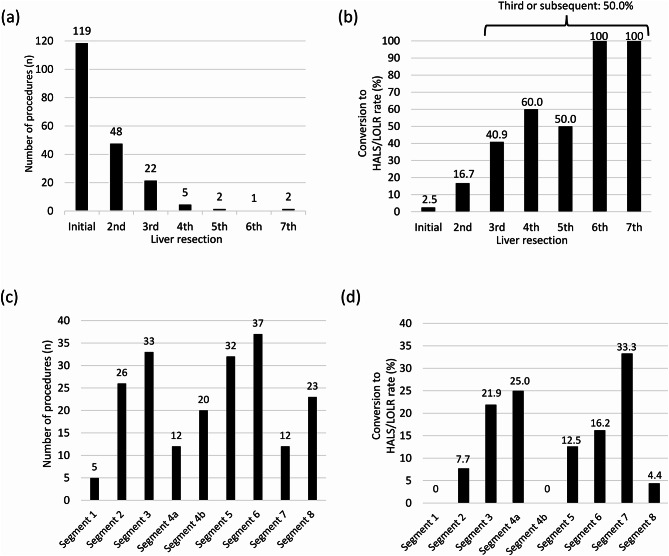
Details of the number of patients and conversion rate for each number of liver resections and resected segments. (**a**) Number of patients for each number of liver resections. (**b**) Conversion rate for each number of liver resections. (**c**) Number of patients for each resected segment. (**d**) Conversion rate for each resected segment. HALS, hand-assisted laparoscopic surgery; OLR, open liver resection.

Details of liver resections and conversion rates by resection number and segment are shown in Fig. 2. Initial liver resection was performed in 119 patients (59.8%), second in 48 patients (24.1%), and third or subsequent in 32 patients (16.1%) (Fig. 2a). The conversion rate for initial liver resection was 2.5% (*n* = 3); for the second, it was 16.7% (*n* = 8), and for third or subsequent, it was 50.0% (*n* = 16) (Fig. 2b). The conversion rate for repeat liver resection was 30.0%. All segments were involved, and the highest conversion rates were observed for resections of segment 7 (33.0%) and segment 4a (25.0%) (Fig. 2c and d).

The conversion group had a significantly higher proportion of males (*P* = 0.012), lower albumin levels (*P* = 0.007), a higher proportion of patients with a history of abdominal surgery (*P* = 0.004) and repeat liver resections (*P* < 0.001).

### Comparison of perioperative outcomes between the pure LLR group and conversion group

The perioperative outcomes are shown in Table 2. Wedge resection was performed in 185 patients (93.0%) and LLS in 10 (5.0%). Four patients (2.0%) were switched to anatomical liver resection, including three segmentectomies and one left lobectomy, for oncology-related reasons. Major postoperative complications occurred in 5 patients (2.5%). One case of bile leakage (0.5%), two cases of intra-abdominal abscess (1.0%), and one case of gastric perforation (0.5%) were successfully treated with percutaneous drainage. One patient in the conversion group (0.5%) required reoperation for a small bowel perforation. No cases of 90-day mortality were observed. Compared with pure LLR, conversion was significantly associated with a longer operative time (*P* = 0.001), increased blood loss (*P* < 0.001), fewer cases in which the Pringle maneuver was possible (*P* = 0.013), and prolonged postoperative hospital stay (*P* = 0.001). There were no significant differences in operative procedures (*P* = 0.358) or the rate of major postoperative complications (*P* = 0.137).

**Table 2:** Comparison of surgical outcomes between pure LLR group and conversion group.

Variables	Whole cohort (*n* = 199)	Pure LLR (*n* = 172)	Conversion to HALS/OLR (*n* = 27)	*P*-value
Operative time (min)	222 (177–279)	217 (173–276)	262 (224–369)	0.001
Blood loss (mL)	55 (20–155)	50 (17–100)	250 (140–530)	< 0.001
Intraoperative blood transfusion	10 (5.0%)	9 (5.2%)	1 (3.7%)	0.735
Implementation of Pringle maneuver	137 (68.8%)	124 (72.1%)	13 (48.1%)	0.013
Operative procedure				0.358
Wedge resection Left lateral segmentectomy Anatomical liver resection	185 (93.0%) 10 (5.0%) 4 (2.0%)	159 (92.4%) 10 (5.8%) 3 (1.7%)	26 (96.3%) 0 (0.0%) 1 (3.7%)	
Simultaneous cholecystectomy	31 (15.6%)	28 (16.3%)	3 (11.1%)	0.491
Resected liver weight (g)	22 (10–40)	22 (10–41)	22 (12–35)	0.452
Any postoperative complication	53 (26.6%)	44 (25.6%)	9 (33.3%)	0.397
Major postoperative complication	5 (2.5%)	3 (1.7%)	2 (7.4%)	0.137
PHLF ≥ ISGLS grade B	0 (0.0%)	0 (0.0%)	0 (0.0%)	NA
Bile leakage ≥ ISGLS grade B	1 (0.5%)	1 (0.6%)	0 (0.0%)	0.691
Reoperation	1 (0.5%)	0 (0.0%)	1 (3.7%)	0.136
Postoperative hospital stay (day)	8 (7–10)	8 (7–10)	10 (8–11)	0.001
90-day mortality	0 (0.0%)	0 (0.0%)	0 (0.0%)	NA

Perioperative outcomes were compared among cases of pure LLR, conversion to HALS, and conversion to OLR (Supplementary Table S1). Compared with pure LLR, conversion to HALS resulted in a significantly longer operative time (*P* = 0.002) and increased blood loss (*P* < 0.002), however, there were no significant differences in postoperative outcomes, including major postoperative complications (*P* = 0.766) and postoperative hospital stay (*P* = 0.996). Conversely, conversion to OLR resulted in significantly greater blood loss (*P* < 0.001) and longer postoperative hospital stay (*P* = 0.001). There were no significant differences between conversion to HALS and OLR.

### The reasons for conversion

The reasons for conversion are summarized in Supplementary Table S2. Of the 27 conversions, the primary cause was failure to progress due to adhesions (*n* = 23; 85.2%). Adhesion-related conversion during the initial liver resection occurred in one case, where trocar insertion was not possible owing to full dense adhesion in the abdominal cavity after treatment for peritonitis. The remaining 22 patients underwent repeat liver resections. Conversions due to bleeding occurred in three patients (11.1%), one of whom required a change of surgical procedure to segmentectomy during the initial wedge resection. One case (3.7%) of initial liver resection was converted to HALS because the lesion could not be exposed.

### Risk factors for conversion

**Fig. 3:**
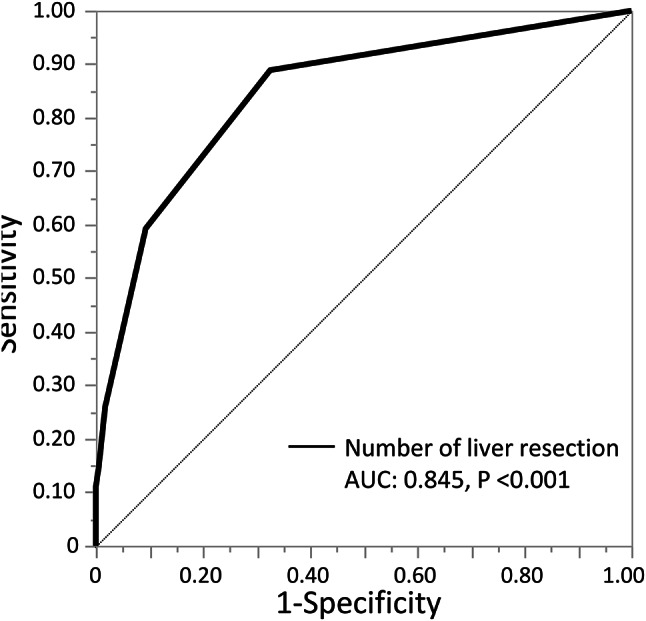
Area under the receiver operating characteristics curve for prediction of conversion for the number of liver resections. AUC, area under the curve; HALS, hand-assisted laparoscopic surgery; OLR, open liver resection.

To define an ideal cut-off value for the number of liver resection, a third liver resection was identified as the cut-off for predicting conversion using ROC curve analysis (area under the curve, 0.845; *P* < 0.001, Fig. 3). Table 3 presents the results of the univariate and multivariate analyses identifying risk factors for conversion.

**Table 3:** Univariate and multivariate analysis for risk factors of conversion to HALS/OLR (*n* = 199).

Variables	Univariate analysis			Multivariate analysis		
	OR	95% CI	*P*-value	OR	95% CI	*P*-value
Age, ≥ 75 (years)	1.166	0.517–2.681	0.517			
Sex, male	5.567	1.579–35.372	0.005	4.546	1.150–30.781	0.029
Body mass index ≥ 30.0 (kg/m^2^)	2.965	0.604–11.409	0.164			
HBV positive	1.830	0.563–5.105	0.294			
HCV positive	1.434	0.576–4.096	0.453			
Diabetes	1.351	0.576–3.077	0.481			
Hypertension	1.710	0.731–4.344	0.221			
Cirrhosis	1.106	0.457–2.970	0.830			
Primary liver malignancy	1.875	0.757–5.333	0.181			
Platelet count < 140 (10^9^/L)	1.631	0.692–3.732	0.257			
Total bilirubin ≥ 0.8 (mg/dL)	1.773	0.782–4.050	0.169			
Albumin < 4.0 (g/dL)	2.935	1.290–6.901	0.010	2.288	0.880–6.055	0.089
Prothrombin time activity < 80 (%)	1.550	0.481–4.254	0.437			
ICG R15 ≥ 15 (%)	2.522	1.111–5.843	0.027	1.636	0.622–4.291	0.315
Aspartate aminotransferase ≥ 35 (IU/L)	1.485	0.574–3.558	0.399			
Alanine aminotransferase ≥ 35 (IU/L)	2.126	0.581–13.718	0.282			
Creatinine ≥ 0.80 (mg/dL)	1.426	0.630–3.250	0.392			
Tumor size ≥ 20 mm	1.285	0.536–2.951	0.564			
Posterosuperior segment	1.225	0.476–2.912	0.660			
Segment 7	3.565	0.895–12.307	0.069			
Difficulty score Intermediate	1.185	0.513–2.911	0.696			
Third or subsequent liver resection	14.182	5.727–36.820	< 0.001	11.220	4.323–30.416	< 0.001
Previous abdominal surgery except for liver resection	1.901	0.831–4.675	0.129			
Previous upper abdominal surgery except for liver resection	1.963	0.522–6.038	0.292			
History of chemotherapy for abdominal disease	1.336	0.418–3.625	0.600			
Surgeon’s experience in LLR < 50 cases	1.875	0.757–5.333	0.181			

In the univariate analysis, male sex, low albumin level, high indocyanine green retention rate at 15 min, and third or subsequent liver resection were significantly associated with conversion. In the multivariate analysis, male sex (odds ratio [OR] = 4.546, 95% confidence interval [CI] = 1.150–30.781; *P* = 0.029) and third or subsequent liver resection (OR = 11.220, 95% CI = 4.323–30.416; *P* < 0.001) emerged as independent risk factors for conversion.

To evaluate the impact of previous liver resection and abdominal surgery on conversion, risk factors for conversion in patients with repeat liver resection (*n* = 80) were analyzed using a Cox regression model (Table 4). A third or subsequent liver resection was confirmed as a significant risk factor for conversion even in repeat liver resections (OR = 5.000, 95% CI = 1.838–14.601; *P* = 0.002). Additionally, previous posterosuperior segment liver resection was identified as a significant risk factor for conversion (OR = 6.111, 95% CI = 1.572–40.620; *P* = 0.007).

**Table 4:** Cox regression analysis for risk factors for conversion to HALS/OLR in repeat liver resection (*n* = 80).

Variables	*n* (%)	OR	95% CI	*P*-value
Third or subsequent liver resections	32 (40.0%)	5.000	1.838–14.601	0.002
Previous posterosuperior segment liver resection	58 (72.5%)	6.111	1.572–40.620	0.007
Previous anterolateral segment liver resection	63 (78.8%)	1.036	0.332–3.625	0.952
Previous liver resection in the same section	27 (33.8%)	2.115	0.782–5.748	0.139
Previous liver resection in the same lobe	58 (72.5%)	1.200	0.416–3.797	0.742
Previous abdominal surgery, except for liver resection	28 (35.0%)	1.457	0.532–4.298	0.470
Previous upper abdominal surgery, except for liver resection	7 (8.8%)	3.533	0.719–19.290	0.118
Previous open abdominal surgery	57 (71.3%)	1.800	0.609–6.112	0.296
Previous open liver resection	53 (66.3%)	1.693	0.621–4.977	0.308

HALS, hand-assisted laparoscopic surgery; CI, confidential interval; OLR, open liver resection; OR, odds ratio.

## Discussion

This multicenter prospective study investigated the risk factors and outcomes of conversion to HALS or OLR in patients who underwent liver resection for tumors indicated for wedge resection or LLS. To the best of our knowledge, this is the first multicenter prospective study to identify the risk factors for conversion in LLR. A notable feature of this study was the laparoscopic approach for all patients scheduled for wedge resection or LLS regardless of previous reported risk factors for conversion, allowing an unbiased evaluation of LLR feasibility. The focus on wedge resection and LLS, —common minor liver resections for benign and small malignant liver tumors—enhanced the accuracy of outcome assessment. In this study, conversion was defined as intraoperative change from pure LLR to HALS or OLR. This definition was adopted because the primary aim of this study was to identify factors associated with the inability to complete pure LLR. Both HALS and OLR represent a failure to continue laparoscopic progress under the predefined safety criteria and therefore shared a common conceptual endpoint in this multicenter prospective study.

Conversion was associated with longer operative time, increased blood loss, and extended postoperative hospital stay compared to pure LLR, consistent with previous studies^4,5^. Furthermore, the conversion group had a high proportion of cases in which the Pringle maneuver was not possible. There were no significant differences in major complications between the groups, likely due to the low incidence of emergency conversions (e.g., massive bleeding). A North American multicenter study highlighted higher risks of complications and mortality in conversions caused by adverse events than conversion for intraoperative findings^19^. In this study, only three conversions were performed emergently due to intraoperative bleeding, and none of these cases resulted in CD grade ≥ 3 postoperative complications. Predefined criteria for conversion likely allowed surgeons to opt for conversion before critical emergencies arose. Because of the small number of emergent events, meaningful statistical comparison between proactive and emergent conversion was not feasible. Nevertheless, the absence of severe postoperative morbidity among patients following emergent conversion suggests that safety thresholds and timely decision-making may have prevented clinical deterioration.

The operative time and blood loss were included in the predefined conversion criteria, and thus the poorer outcomes for these parameters in the conversion group may have slightly influenced the association based on this definition. However, prolonged adhesion lysis, difficulty maintaining an adequate surgical field, and increased blood loss reflect clinically meaningful thresholds by which surgeons should appropriately decide not to continue pure LLR to ensure surgical safety. Therefore, we consider that the observed differences largely represent the practical consequences of intraoperative conversion rather than solely an artifact of definition. Although the results comparing intraoperative factors may contain slight artifacts based on safety criteria, they are considered to primarily represent the practical outcomes of intraoperative conversion.

The major cause of conversion in this study was adhesions (88.9%). Excluding patients with anatomical liver resection, which often involves tumors close to major vessels contributed to the relatively low bleeding-related conversion rate (11.1%). A meta-analysis of 25 studies reported LLR conversion rates ranging from 4% to 30.5%, with an average of 12.2%^20^. The conversion rate of 13.6% in this study, despite excluding anatomical liver resections, reflects the inclusion of HALS as a conversion and the laparoscopic approach for all patients. Kawaguchi et al. reported a 1.7% conversion rate for initial wedge resection and LLS, similar to the 2.5% rate observed in this study, showing no marked difference^21^. In contrast, the 30.0% conversion rate for repeat liver resection in this study exceeded previously reported rates (up to 17.0%)^22^. Patients with a history of two or more liver resections or major liver resections may have been excluded from the indications for LLR in previous retrospective studies due to a predicted high conversion rate. The lack of prior data on the number of previous liver resections suggests that this higher rate of repeat procedures is a notable finding.

A third or subsequent liver resection and male sex were identified as independent risk factors for conversion in the multivariate analysis. Two large-scale studies demonstrated that previous liver resection is a significant risk factor for conversion^12,19^. A European retrospective analysis of 2,861 cases found no association between previous upper abdominal surgery and conversion, suggesting that adhesions from previous liver resections, rather than other upper abdominal surgeries, increase the likelihood of conversion^10^. This aligns with the present study, which found no link between previous abdominal surgery (excluding liver resections) and conversion risk. Regardless of the history of laparotomy, dense adhesions around the liver and hepatic hilum after multiple resections likely contribute to this increased risk. In this study, ROC analysis identified a third or subsequent liver resections as the cut-off point for predicting conversion. Although repeat LLRs have become increasingly common and effective^23^, no previous report has detailed the influence of the number of previous liver resections. Even in the subgroup analysis of repeat liver resection, a third or subsequent resection remained a significant risk factor. However, it should be noted that the confidence intervals of the odds ratios were relatively wide, indicating limited precision of these estimates. This may reflect the relatively small number of conversion events in this prospective cohort. Therefore, the findings of this study should be interpreted with caution, and validation in larger-scale studies is needed.

Furthermore, previous posterosuperior segment liver resection emerged as a significant risk factor for conversion during repeat liver resection. The posterosuperior segment is one of the most difficult parts of the liver to resect, requiring extensive liver mobilization, frequent anatomical resections, and a larger parenchymal dissection area, all of which increase operative complexity and blood loss^24^. These factors may contribute to the formation of extensive adhesions, increasing the risk of conversion. Interestingly, previous resection of the same segment or lobe and previous open liver resection were not risk factors for conversion in repeat LLR. Instead, adhesions resulting from previous posterosuperior segments or multiple resections appear more likely to affect areas near the resection site, increasing the likelihood of conversion.

In this study, male sex was also associated with conversion, consistent with findings from multiple international multicenter studies and one meta-analysis^12,25^. Wang et al. suggested that this is because male patients have a larger liver volume, necessitating greater parenchymal transection and a higher incidence of liver disease^26,27^. The higher conversion rate in male patients may be partly explained by sex-related anatomical and physiological differences, such as larger liver size and greater visceral fat accumulation, which can restrict the operative field and make laparoscopic manipulation more challenging. These factors could contribute to longer operative time and intraoperative difficulty, thereby increasing the likelihood of conversion to HALS or OLR^26,27^. Additionally, males exhibit a higher recurrence risk after surgery for liver cancer^28^, potentially increasing the likelihood of repeat liver resections and subsequent conversion. In the present study, male patients had a significantly higher proportion of repeat liver resection than female patients (48.6% vs. 18.2%, *P* < 0.001, Supplementary Table S3). Although LLR is effective regardless of sex, it is necessary to consider male-specific liver characteristics, and history of liver resection in planning LLR.

Although this study focused on wedge resection and LLS, these procedures represent the most standardized and reproducible settings for evaluating the fundamental risk factors of conversion to HALS or OLR. Parenchymal or major liver resections, additional technical challenges including proximity to major vessels and larger parenchymal transection area are known to increase the likelihood of conversion^29,30^. Therefore, the present findings should be interpreted as baseline risk factors that may coexist with anatomical and major LLR. To improve LLR safety, several scoring systems have been proposed to evaluate surgical difficulty, focusing on procedure type, tumor characteristics, and liver function^2,21,31,32^. Although Halls et al. incorporated OLR as a predictive factor for intraoperative complications^32^, these scores often do not include patient-specific characteristics, such as the number of previous liver resections or abdominal disease history. Based on the results of this study, third or subsequent liver resection should be used to the preoperative evaluation as a highly significant risk factor for conversion in LLR. In addition, when assessing the difficulty of repeat LLR, previous posterosuperior liver resection should be considered as a significant risk factor for conversion. In wedge resection and LLS, the location of the tumor may not cause a significant change in difficulty that affects conversion. Given the negative impact of conversion on perioperative outcomes, a more detailed assessment of the difficulty of LLR is highly effective. Although third or subsequent liver resections and male sex were independent risk factors for conversion in this study, they should not be considered contraindications to performing LLR. We believe these risk factors may serve not as components of a formalized difficulty scoring system, but as predictors of technical challenges including severe adhesions, limited liver mobility, or difficulty securing an adequate surgical field. In patients with these factors who are at a high risk for conversion, strategic surgical planning, including preparing for the possibility of early conversion, ensuring availability of additional personnel or equipment and selecting optimal trocar placement to account for restricted liver mobility, is essential.

In recent years, robotic liver resection has become increasingly widespread, providing the freedom of manipulation with articulated forceps and dimensional visualization compared with conventional LLR. Robotic liver resection has been reported to be superior to LLR in terms of decreased operative time, decreased blood loss and a reduced open conversion rate^24,33^. However, the availability of robotic systems remains limited by high acquisition and maintenance costs, institutional resources, and local social situations^34^. In addition, the lack of tactile feedback and dependence on visual cues are considered disadvantages of LLR and robotic surgery^35^. Even in high-volume centers, conversion to HALS or OLR continues to play an important role, particularly in situations such as emergency procedures or cases requiring rapid tactile control for hemostasis^7,13^. Accordingly, identifying risk factors for conversion remains clinically relevant in the current era of diversified minimally invasive liver surgery. While patients predicted to have a high risk of conversion may benefit from preparation for HALS or OLR, those without risk factors may be suitable candidates for a robot-assisted approach to minimize the likelihood of intraoperative conversion.

This study has several limitations. First, selection bias cannot be completely excluded since this was not a randomized trial; however, there are no previous prospective trials for the identification of conversion risk in LLR, and our data provides more practical information. Second, perioperative outcomes were not compared with a control group that underwent planned open liver resection. Whether conversion to HALS or OLR itself was the cause of worsening perioperative outcomes cannot be clearly evaluated. Third, this study was conducted with patients scheduled for wedge resection and LLS, limiting the generalizability of the findings to major or anatomical LLR. This study included institutions other than high-volume centers and was designed to assess surgical procedures generally performed for isolated small tumors. Further studies are needed to evaluate whether the results of this study apply to major or anatomical LLR.

In conclusion, this study identified third or subsequent liver resection and male sex as significant risk factors for conversion during LLR. Patients requiring conversion experienced longer operative times, greater blood loss, and extended postoperative hospital stays.

## Supplementary Information

Below is the link to the electronic supplementary material.

Supplementary Material 1

## Data Availability

The data that supports the findings of this study are available from the corresponding author upon reasonable request.
