# Supplementary material for: Risk factors of conversion to hand-assisted laparoscopic surgery or open surgery in laparoscopic liver resection: a multicenter prospective study (HiSCO-08)
Source: Sci Rep. 2025 Dec 26;16:3873. doi: 10.1038/s41598-025-34013-3 (PMC12852719; doi:10.1038/s41598-025-34013-3)
Supplement: Supplementary file 1 — Supplementary Material 1 [file 41598_2025_34013_MOESM1_ESM.pdf]

Supplementary Table S1. Comparison of surgical outcomes between pure LLR group, conversion to HALS group and conversion to OLR group

Variables	Pure LLR (n = 172)	Conversion to HALS (n = 5)	Conversion to OLR (n = 22)	P-value (LLR vs HALS)	P-value (LLR vs OLR)	P-value (HALS vs OLR)
Operative time (min)	217 (173–276)	369 (260–471.5)	250.5 (220–267)	0.002	0.084	0.076
Blood loss (mL)	50 (17–100)	386 (110–718.5)	250 (151–580)	< 0.001	< 0.001	0.997
Intraoperative blood transfusion	9 (5.2%)	0 (0.0%)	1 (4.6%)	0.600	0.891	0.627
Implementation of Pringle maneuver	124 (72.1%)	2 (40.0%)	11 (50.0%)	0.145	0.034	0.686
Operative procedure				0.408	0.361	0.627
Wedge resection	159 (92.4%	5 (100.0%)	21 (95.5%)			
Left lateral segmentectomy	10 (5.8%)	0 (0.0%)	0 (0.0%)			
Anatomical liver resection	3 (1.7%)	0 (0.0%)	1 (4.5%)			
Concurrent cholecystectomy	28 (16.3%)	1 (20.0%)	2 (9.1%)	0.825	0.380	0.484
Resected liver weight (g)	22 (10–41)	19 (13–43.5)	24 (12–35)	0.877	0.808	0.986
Any postoperative complication	44 (25.6%)	1 (20.0%)	8 (36.4%)	0.778	0.282	0.484
Major postoperative complication	3 (1.7%)	0 (0.0%)	2 (9.1%)	0.766	0.099	0.484
PHLF ≥ ISGLS grade B	0 (0.0%)	0 (0.0%)	0 (0.0%)	NA	NA	NA
Bile leakage ≥ ISGLS grade B	1 (0.6%)	0 (0.0%)	0 (0.0%)	0.864	0.720	NA
Reoperation	0 (0.0%)	0 (0.0%)	1 (4.5%)	NA	0.113	0.627
Postoperative hospital stay (day)	8 (7–10)	9 (7–10.5)	10 (9–12)	0.996	0.001	0.174
90-day mortality	0 (0.0%)	0 (0.0%)	0 (0.0%)	NA	NA	NA

Variables are expressed as median (interquartile range) or number (%)
HALS, hand-assisted laparoscopic surgery, ISGLS, international study group of liver surgery; LLR, laparoscopic liver resection; OLR, open liver resection; PHLF, post-hepatectomy liver failure

Supplementary Table S2. Reasons for conversion

Variables	Conversion (n = 27)
Adhesions	23 (85.2%)
Bleeding	3 (11.1%)
Inability to expose the lesion	1 (3.7%)

Variables are expressed as number (%)

Supplementary Table S3: Comparison of patient backgrounds between male and female

Variables	Male (n = 144)	Female (n = 55)	P-value
Age (years)	75 (70–80)	73 (66–80)	0.054
Body mass index (kg/m^2)	23.9 (21.5–26.4)	22.8 (21.1–26.7)	0.637
HBV positive	27 (18.8%)	4 (7.3%)	0.033
HCV positive	37 (25.7%)	19 (34.5%)	0.220
Diabetes	55 (38.1%)	14 (25.5%)	0.086
Hypertension	92 (63.9%)	27 (49.1%)	0.058
Cirrhosis	38 (26.3%)	17 (30.9%)	0.527
Diagnosis			0.020
Primary liver malignancy	103 (71.5%)	31 (56.3%)	
Liver metastasis	38 (26.4%)	18 (32.7%)	
Benign tumor	3 (2.1%)	6 (10.9%)	
Total bilirubin (mg/dL)	0.8 (0.6–1.1)	0.7 (0.5–1.1)	0.781
Albumin (g/dL)	4.1 (3.8–4.3)	4.0 (3.7–4.4)	0.922
Prothrombin time activity (%)	96.9 (87.3–104.0)	99 (92.3–105.3)	0.388
Child-Pugh classification A/B	139 (96.5%)/5 (3.5%)	53 (96.4%)/2 (3.6%)	0.955
Platelet count (10^9/L)	17.5 (13.8–21.2)	17.0 (12.8–21.9)	0.922
Aspartate aminotransferase (IU/L)	24 (20–34)	24 (20–35)	0.663
Alanine aminotransferase (IU/L)	19.5 (15–28)	18 (12–27)	0.511
ICG R15 (%)	13.2 (8.5–18.5)	9.4 (6.5–15.0)	0.200
Creatinine (mg/dL)	0.88 (0.78–1.06)	0.65 (0.58–0.78)	0.003
Tumor size (mm)	15 (10–22)	15 (10–20)	0.485
Tumor location Posterosuperior segments	39 (27.1%)	13 (23.6%)	0.618
Difficulty score Low/Intermediate	58 (40.3%)/86 (59.7%)	40 (72.7%)/15 (27.3%)	0.008
Previous abdominal surgery	111 (77.1%)	35 (63.6%)	0.060
Previous upper abdominal surgery	82 (56.9%)	13 (23.6%)	0.007
Initial/Repeat liver resection	70 (48.6%)	45 (81.8%)	<0.001
Third or subsequent liver resection	27 (18.8%)	5 (9.0%)	0.082
Surgeon's experience with LLR < 50 cases	98 (68.1%)	35 (63.6%)	0.556

Variables are expressed as median (interquartile range) or number (%)

HBV, hepatitis B virus; HCV, hepatitis C virus; ICG R15, indocyanine green retention rate at 15 minutes; LLR, laparoscopic liver resection
